# Impact of lockdowns on paediatric asthma hospital presentations over three waves of COVID-19 pandemic

**DOI:** 10.1186/s13223-022-00691-1

**Published:** 2022-06-16

**Authors:** Nusrat Homaira, Nan Hu, Louisa Owens, Mei Chan, Melinda Gray, Philip N Britton, Hiran Selvadurai, Raghu Lingam, Adam Jaffe

**Affiliations:** 1 Discipline of Paediatrics and Child Health, School of Clinical Medicine, UNSW Sydney, Randwick, Australia; 2 Sydney Children’s Hospital, Randwick, Australia; 3 National Centre for Immunisation Research and Surveillance, Westmead, Australia; 4 The Children’s Hospital at Westmead, Westmead, Australia; 5 Sydney Medical School, University of Sydney, Sydney, Australia

**Keywords:** COVID-19, Lockdowns, Paediatric asthma

## Abstract

Public health measures to mitigate the COVID-19 pandemic have altered health care for chronic conditions. The impact of the COVID-19 pandemic on paediatric asthma, the most common chronic respiratory cause of childhood hospitalisation, in Australia, remains unknown. In a multicentre study, we examined the impact of three waves of COVID-19 on paediatric asthma in New South Wales Australia. Time series analysis was performed to determine trends in asthma hospital presentations in children aged 2–17 years before (2015–2019) and during the COVID-19 pandemic (2020–2021) using emergency department and hospital admission datasets from two large tertiary paediatric hospitals.

In this first report from Australia, we observed a significant decrease in asthma hospital presentations during lockdown periods including April (68.85%), May (69.46%), December (49.00%) of 2020 and August (66.59%) of 2021 compared to pre-pandemic predictions.

The decrease in asthma hospital presentations coincided with the lockdown periods during first, second and third waves of the COVID-19 pandemic and was potentially due to reduced transmission of other common respiratory viruses from restricted movement.

## Background

Asthma is one of the most common chronic respiratory conditions of childhood affecting almost 14% of children worldwide [1]. Children with asthma are at risk of frequent hospital presentations due to acute asthma attacks often caused by viral respiratory infections and exposures to increased levels of pollen and air pollution [2–4]. Some reports have indicated a decrease in paediatric asthma hospital presentation associated with the initial wave of the COVID-19 pandemic [5]. Several factors have been linked to this observed decrease including a decrease in transmission of respiratory infection due to school closures and less exposure to outdoor air pollution from staying at home [6]. However these reports have been based on single centre and captured the initial wave of the pandemic.

In New South Wales (NSW) Australia, which is the most densely populated state of the country, as of September 2021, we have experienced three waves of the COVID-19 pandemic. The first wave of the COVID-19 pandemic in New South Wales (NSW) Australia was during February–March 2020 which prompted a state-wide lockdown from March 2020 including border and school closures and stay at home orders. Schools returned to face-to-face learning from mid-May in NSW but restrictions on social gatherings were still in place. There was a resurgence of COVID-19 cases during October and December of 2020 which again resulted in restricted movement within greater NSW and introduction of mask mandates in indoor settings and on public transportation. The third wave of the COVID-19 pandemic in NSW due to the emergence of the Delta variant of SARS-CoV-2 resulted in wider lockdowns in greater Sydney on 26th June 2020, with mandatory mask use in indoor settings and on public transport and with schools going back to remote learning. Additionally, in an effort to contain the highly transmissible Delta strain, some areas in Sydney with increasing number of cases went under curfew effective between 9 pm and 5am from 23rd August 2021. Mandatory mask use while in outdoor settings was also put in place in NSW from the same date. As of September 30, 2021, greater Sydney remained in lockdowns, with easing of curfew and stay-at-home order to be lifted from 11 October and schools resuming face-to-face learning from end of October 2021.

The COVID-19 lockdowns led to generalised decrease in healthcare resource utilisation for common chronic conditions. The impact of COVID-19 lockdowns on paediatric asthma in Australia remains unclear. We aimed to examine the impact of lockdowns associated with three waves of the COVID-19 pandemic on paediatric asthma hospital presentations in NSW, Australia.

## Methods

In this multicentre study, we analysed data from the Sydney Children’s Hospitals Network (SCHN) in NSW, the largest provider of tertiary paediatric services in Australia, comprising two large hospitals including the Sydney Children’s Hospital at Randwick and the Children’s Hospital at Westmead. We included the SCHN’s electronic medical records from 1st January 2015 to 31st August 2021. Data were extracted from two routinely collected datasets including hospital admission and emergency department (ED) attendance data for hospital presentations associated with asthma (predominantly allergic asthma J45.0, non-allergic asthma J45.1, mixed asthma J45.8, asthma unspecified J45.9 and status asthmaticus J46) in children aged 2–17 years, using International Classification of Diseases 10th revision Australian Modification for hospital admissions and Systemised Nomenclature of Medicine Clinical Terms for ED attendances. We included children aged ≥ 2 years of age as asthma diagnosis is challenging in younger children.

We compared asthma hospital presentations (inpatient admissions and ED attendances) in the pandemic period (1st January 2020–31st August 2021) with the pre-pandemic period (1st January 2015–31st December 2019) and plotted counts of asthma hospital presentation by months and performed time series analysis to predict frequencies and their 95% confidence intervals (CIs) in 2020–2021 based on pre-pandemic years, using autocorrelation error and autoregressive integrated moving average (ARIMA) models. We estimated the percentage difference between the observed and the predicted frequencies in 2020 and 2021 compared to pre-pandemic years and stratified the analysis by age groups: 2–5 years, 6–12 years and 13–17 years. This study was approved by the SCHN Human Research Ethics Committee (2020/ETH01432).

## Results

In the pre-pandemic years (2015–2019) there were in total 492,863 hospital presentations in children aged 2–17 years, of these 13,160 (2.67%) were due to asthma and in pandemic years (2020–2021) there were 163,521 hospital presentations of which 3364 (2.05%) were due to asthma (Table 1).

**Table 1:** All cause and asthma related paediatric inpatient admissions and emergency department attendances in children aged 2–17 years across Sydney Children’s Hospitals Network in pre-pandemic (2015–2019) and pandemic periods (2020–2021)

Year of presentation																
	2015		2016		2017		2018		2019		2015–2019		2020		2021 (up to 31st August	
	N	%	N	%	N	%	N	%	N	%	N	%	N	%	N	%
Any diagnosis																
2–17 years (Total)	96,431	100	97,961	100	98,091	100	95,439	100	104,940	100	492,863	100	93,818	100	69,703	100
2–5 years	40,860	42.37	41,431	42.29	40,230	41.01	38,909	40.76	43,269	41.23	204,700	41.53	36,490	38.89	28,675	41.3
6–12 years	39,202	40.65	39,737	40.56	40,325	41.10	38,903	40.76	42,596	40.59	200,763	40.73	37,469	39.94	26,785	38.42
13–17 years	16,369	16.97	16,793	17.14	17,536	17.87	17,627	18.46	19,075	18.17	87,400	17.73	19,859	21.17	14,243	20.04
Asthma hospital presentations																
2–17 years (Total)	3078	3.19	2665	2.72	2540	2.58	2295	2.40	2582	2.46	13,160	2.67	1912	2.03	1452	2.08
2–5 years	1640	53.28	1262	47.35	967	38.07	878	38.25	848	32.84	5595	42.51	601	31.43	440	30.30
6–12 years	1238	40.22	1158	43.45	1234	48.58	1062	46.27	1262	48.87	5954	45.24	977	51.09	737	50.75
13–17 years	200	6.49	245	9.19	339	13.34	355	15.46	472	18.28	1611	12.24	334	17.46	275	18.93

In 2020–2021, the overall percentage difference in the annual observed number of asthma hospital presentations were not different compared with pre-pandemic years. However, the observed frequency of asthma hospital presentations in April, May and December of 2020 and August 2021 were significantly lower than predicted numbers based on the trend of these months observed in 2015–19 (68.85% reduction in April, 69.46% in May and 49% in December of 2020 and 66.59% in August of 2021; p < 0.05) (Table 2). The reduction in asthma hospital presentation in April–May of 2020 and August 2021 was observed across all the age-groups excluding children aged 2–5 years which could have been due to very small numbers of observed hospital presentations in this age-group.

**Table 2:** Differences between observed and predicted numbers of paediatric asthma inpatient admissions and emergency department attendances by months in 2020 and 2021 compared to pre-pandemic years (2015–2019)

Month	Number of inpatient admissions and ED attendances					
	Observed number	Predicted number (95%CI)	Percentage difference from predicted number (%) (95% CI)	Observed number	Predicted number (95% CI)	Percentage difference from predicted number (%) (95% CI)
	Year 2020			Year 2021 (up to 31st August)		
All children aged 2–17 years						
All year	1912	2223.4 (1230.58, 3216.22)	−14.01 (−40.55, 55.37)	1452	2087.2 (940.79, 3233.61)	−30.43 (−55.1, 54.34)
JAN	124	139.88 (53.63, 226.14)	−11.35 (−45.17, 131.23)	73	143.27 (32.14, 254.41)	−49.05 (−71.31, 127.14)
FEB	227	267.78 (181.48, 354.07)	−15.23 (−35.89, 25.08)	234	240.59 (129.35, 351.83)	−2.74 (−33.49, 80.91)
MAR	244	245.44 (159.11, 331.78)	−0.59 (−26.46, 53.35)	254	223.34 (111.99, 334.68)	13.73 (−24.11, 126.8)
APR	**60**	**192.61 (106.23, 278.98)**	−68.85 (−78.49, −43.52) ^**^	186	182.83 (71.38, 294.28)	1.73 (−36.79, 160.59)
MAY	**76**	**248.82 (162.41, 335.24)**	−69.46 (−77.33, −53.2) ^***^	337	225.48 (113.92, 337.04)	49.46 (−0.01, 195.82)
JUN	211	199.03 (112.58, 285.49)	6.01 (−26.09, 87.43)	216	187.29 (75.63, 298.96)	15.33 (−27.75, 185.61)
JUL	165	137.81 (51.31, 224.31)	19.73 (−26.44, 221.56)	79	140.39 (28.61, 252.16)	−43.73 (−68.67, 176.12)
AUG	198	240.54 (154, 327.08)	−17.69 (−39.46, 28.57)	**73**	**218.51 (106.62, 330.4)**	−66.59 (−77.91, −31.53) ^***^
SEP	163	189.23 (102.65, 275.81)	−13.86 (−40.9, 58.8)			
OCT	134	153.17 (66.54, 239.8)	−12.52 (−44.12, 101.37)			
NOV	208	210.91 (124.24, 297.58)	−1.38 (−30.1, 67.42)			
DEC	**102**	200.01 (113.3, 286.73)	**−49 (−64.43, −9.97)** ^*^			
Children aged 2–5 years						
All year	601	528.6 (−119.32, 1176.52)	–	440	331.8 (−416.35, 1079.95)	–
JAN	41	38.39 (−11.82, 88.59)	–	16	26.5 (−36.29, 89.29)	–
FEB	68	82.93 (32.7, 133.17)	−18.01 (−48.94, 107.96)	83	56.89 (−5.98, 119.76)	–
MAR	90	68.66 (18.39, 118.92)	31.09 (−24.32, 389.37)	88	46.57 (−16.37, 109.52)	–
APR	9	55.07 (4.77, 105.37)	−83.66 (−91.46, 88.5)	65	36.74 (−26.29, 99.76)	–
MAY	8	57.4 (7.07, 107.73)	−86.06 (−92.57, 13.08)	101	37.91 (−25.19, 101.02)	–
JUN	70	40.36 (−10, 90.72)	–	61	25.68 (−37.51, 88.87)	–
JUL	51	27.46 (−22.93, 77.86)	–	12	16.32 (−46.95, 79.59)	–
AUG	59	72.7 (22.28, 123.13)	−18.85 (−52.08, 164.83)	14	47.19 (−16.16, 110.55)	–
SEP	49	46.66 (−3.8, 97.12)	–			
OCT	62	33.77 (−16.73, 84.26)	–			
NOV	67	44.4 (−6.12, 94.93)	–			
DEC	27	52.27 (1.71, 102.84)	−48.35 (−73.74, 1478.79)			
Children aged 6–11 years						
All year	977	1176.4 (742.15, 1610.65)	−16.95 (−39.34, 31.64)	737	1171.6 (670.17, 1673.03)	−37.09 (−55.95, 9.97)
JAN	50	68.3 (17.98, 118.62)	−26.79 (−57.85, 178.11)	39	79.32 (16.95, 141.68)	−50.83 (−72.47, 130.06)
FEB	123	133 (82.64, 183.35)	−7.52 (−32.91, 48.83)	113	122.92 (60.48, 185.36)	−8.07 (−39.04, 86.85)
MAR	114	124.24 (73.86, 174.62)	−8.24 (−34.71, 54.35)	131	117.02 (54.5, 179.54)	11.95 (−27.04, 140.37)
APR	**30**	**96.61 (46.2, 147.02)**	−68.95 (−79.6, −35.06) ^*^	87	98.41 (35.81, 161.01)	−11.59 (−45.97, 142.98)
MAY	**50**	**128.29 (77.84, 178.73)**	−61.02 (−72.03, −35.77) ^**^	178	119.76 (57.07, 182.44)	48.64 (−2.43, 211.87)
JUN	110	112.12 (61.64, 162.59)	−1.89 (−32.35, 78.46)	116	108.86 (46.1, 171.62)	6.56 (−32.41, 151.63)
JUL	79	77.08 (26.57, 127.59)	2.49 (−38.08, 197.35)	39	85.25 (22.41, 148.1)	−54.25 (−73.67, 74.06)
AUG	110	113.47 (62.93, 164.01)	−3.06 (−32.93, 74.8)	**34**	**109.78 (46.85, 172.71)**	−69.03 (−80.31, −27.43) ^*^
SEP	82	98.65 (48.07, 149.22)	−16.88 (−45.05, 70.58)			
OCT	56	79.11 (28.5, 129.72)	−29.21 (−56.83, 96.51)			
NOV	117	117.52 (66.88, 168.17)	−0.44 (−30.43, 74.95)			
DEC	56	101.35 (50.67, 152.03)	−44.75 (−63.17, 10.52)			
Children aged 12–17 years						
All year	**334**	**518.4 (401.75, 635.05)**	**−35.57 (−47.41, −16.86)** ^**^	**275**	**583.8 (449.11, 718.49)**	−52.89 (−61.73, −38.77) ^**^
JAN	33	39.61 (27.34, 51.88)	−16.68 (−36.39, 20.71)	**18**	**45.92 (32.27, 59.57)**	**−60.8 (−69.78, −44.22)** ^***^
FEB	36	42.63 (30.35, 54.92)	−15.56 (−34.45, 18.62)	38	47.38 (33.71, 61.06)	−19.8 (−37.77, 12.74)
MAR	40	44.48 (32.18, 56.78)	−10.08 (−29.55, 24.29)	35	48.39 (34.68, 62.09)	−27.67 (−43.63, 0.92)
APR	**21**	**41.22 (28.91, 53.54)**	**−49.06 (−60.77, −27.37)** ^**^	34	47.38 (33.65, 61.12)	−28.25 (−44.37, 1.04)
MAY	**18**	**50.53 (38.21, 62.86)**	**−64.38 (−71.37, −52.89)** ^***^	58	51.32 (37.55, 65.08)	13.02 (−10.88, 54.46)
JUN	**31**	**44.13 (31.79, 56.48)**	**−29.76 (−45.11, −2.49)** ^*^	39	49.08 (35.28, 62.88)	−20.54 (−37.97, 10.53)
JUL	35	40.48 (28.13, 52.84)	−13.55 (−33.76, 24.43)	**28**	**47.92 (34.1, 61.75)**	**−41.57 (−54.66, −17.88)** ^**^
AUG	**29**	**46.65 (34.28, 59.02)**	**−37.84 (−50.87, −15.4)** ^**^	**25**	**50.62 (36.76, 64.48)**	**−50.61 (−61.23, −32)** ^**^
SEP	32	43.79 (31.4, 56.17)	−26.92 (−43.03, 1.91)			
OCT	**16**	**44.06 (31.66, 56.47)**	**−63.69 (−71.67, −49.46)** ^***^			
NOV	**24**	**45.52 (33.1, 57.94)**	**−47.27 (−58.58, −27.49)** ^***^			
DEC	**19**	**45.01 (32.57, 57.45)**	**−57.79 (−66.93, −41.67)** ^***^			

The numbers in bold highlight significant differences *p < 0.05

^**^p < 0.01

^***^p < 0.001

## Discussion

In this first report from Australia, we have shown significant reductions in paediatric asthma hospital presentations during April, May and December of 2020 and August of 2021 which coincided with the periods of restricted movement within NSW due to measures implemented to mitigate the three waves of the COVID-19 pandemic in NSW. We observed a reduction of 50–70% in paediatric asthma hospital presentations which is comparable to reductions observed during the lockdowns implemented in the initial stages of the pandemic in other parts of the world [5].

There are several possible explanations for this observed pattern. Firstly a reduction in paediatric hospital presentations associated with viral respiratory infections was also observed during April and May 2020 in NSW [7]. Viral respiratory infections are common triggers for asthma attacks. Restrictions on face-to-face learning during the lockdown periods may have reduced transmission of respiratory viruses within school settings. Indeed peaks in asthma hospital presentations in children are associated with return to school [8].

The observed reduced number of asthma hospital presentations during April, May of 2020 and August 2021 could also be linked to reduced exposure to outdoor air pollution from stay-at home orders and children staying indoors. There is evidence that general outdoor air quality in NSW improved during the lockdown period [9]. Additionally October–December coincides with major grass pollen peaks in NSW and limited outdoor movement during the second wave of the COVID-19 pandemic could help explain the reduced asthma hospital presentations during these months in children aged 12–17 years who generally have higher mobility compared to younger children.

In response to disruptions to health services due to lockdowns, the Australian government enhanced telehealth to enable access to routine healthcare services via telephone or videoconferencing. It is also possible that general fear within community residents about contracting COVID-19 which may have led to reduced physical visits to hospitals and opting for telehealth services. We could not look into adherence to asthma medications during lockdown periods. There are reports of increased purchase of asthma inhaler medications during lockdown period which may lead to improved self-management of asthma symptoms [10].

Our data demonstrated that during the three waves of the COVID-19 pandemic in NSW so far, measures to contain the pandemic including lockdowns, mask mandates and restricted outdoor movement may have led to a reduction in paediatric asthma hospital presentations. Chronic conditions constitute a major burden on the health system. Healthcare utilisation associated with chronic conditions declined globally during the pandemic. While this decline has been associated with lockdowns, such an approach is not feasible or sustainable in the absence of an infectious disease outbreak. Therefore further research to determine the positive factors associated with this observed pattern could help develop strategies to mitigate the burden of chronic conditions such as asthma on the health system.

## Data Availability

The data that support the findings of this study is available upon appropriate ethics approval.

## References

[1] Global Asthma Network. The Global Asthma Report 2014. Auckland: Global Asthma Network (GAN) Steering Group, 2014.

[2] Khetsuriani N, Kazerouni NN, Erdman DD, Lu X, Redd SC, Anderson LJ (2007). Prevalence of viral respiratory tract infections in children with asthma. J Allergy Clin Immunol.

[3] Osborne NJ, Alcock I, Wheeler BW, Hajat S, Sarran C, Clewlow Y (2017). Pollen exposure and hospitalization due to asthma exacerbations: daily time series in a European city. Int J Biometeorol.

[4] Bouazza N, Foissac F, Urien S, Guedj R, Carbajal R, Tréluyer J-M (2018). Fine particulate pollution and asthma exacerbations. Arch Dis Child.

[5] Krivec U, Kofol Seliger A, Tursic J (2020). COVID-19 lockdown dropped the rate of paediatric asthma admissions. Arch Dis Child.

[6] Oreskovic NM, Kinane TB, Aryee E, Kuhlthau KA, Perrin JM (2020). The unexpected risks of COVID-19 on asthma control in children. J Allergy Clin Immunol Pract.

[7] Britton PN, Hu N, Saravanos G, Shrapnel J, Davis J, Snelling T (2020). COVID-19 public health measures and respiratory syncytial virus. Lancet Child Adolesc Health.

[8] Sears MR, Johnston NW (2007). Understanding the September asthma epidemic. J Allergy Clin Immunol.

[9] Duc H, Salter D, Azzi M, Jiang N, Warren L, Watt S (2021). The Effect of lockdown period during the COVID-19 pandemic on air quality in Sydney region, Australia. Int J Environ Res Public Health.

[10] Davies GA, Alsallakh MA, Sivakumaran S, Vasileiou E, Lyons RA, Robertson C (2021). Impact of COVID-19 lockdown on emergency asthma admissions and deaths: national interrupted time series analyses for Scotland and Wales. Thorax.

